# CBRAIN: a web-based, distributed computing platform for collaborative neuroimaging research

**DOI:** 10.3389/fninf.2014.00054

**Published:** 2014-05-21

**Authors:** Tarek Sherif, Pierre Rioux, Marc-Etienne Rousseau, Nicolas Kassis, Natacha Beck, Reza Adalat, Samir Das, Tristan Glatard, Alan C. Evans

**Affiliations:** ^1^ACElab, McConnell Brain Imaging Centre, Montreal Neurological Institute, McGill UniversityMontreal, QC, Canada; ^2^CREATIS, INSERM, Centre National de la Recherche Scientifique, Université de LyonLyon, France

**Keywords:** eScience, distributed computing, meta-scheduler, collaborative platform, interoperability, cloud computing, neuroimaging, visualization

## Abstract

The Canadian Brain Imaging Research Platform (CBRAIN) is a web-based collaborative research platform developed in response to the challenges raised by data-heavy, compute-intensive neuroimaging research. CBRAIN offers transparent access to remote data sources, distributed computing sites, and an array of processing and visualization tools within a controlled, secure environment. Its web interface is accessible through any modern browser and uses graphical interface idioms to reduce the technical expertise required to perform large-scale computational analyses. CBRAIN's flexible meta-scheduling has allowed the incorporation of a wide range of heterogeneous computing sites, currently including nine national research High Performance Computing (HPC) centers in Canada, one in Korea, one in Germany, and several local research servers. CBRAIN leverages remote computing cycles and facilitates resource-interoperability in a transparent manner for the end-user. Compared with typical grid solutions available, our architecture was designed to be easily extendable and deployed on existing remote computing sites with no tool modification, administrative intervention, or special software/hardware configuration. As October 2013, CBRAIN serves over 200 users spread across 53 cities in 17 countries. The platform is built as a generic framework that can accept data and analysis tools from any discipline. However, its current focus is primarily on neuroimaging research and studies of neurological diseases such as Autism, Parkinson's and Alzheimer's diseases, Multiple Sclerosis as well as on normal brain structure and development. This technical report presents the CBRAIN Platform, its current deployment and usage and future direction.

## Introduction

For the past decade, scientists in all fields of research have had to cope with the effects of accelerated data acquisition and accumulation, large increases in study size and required computational power (Bell et al., 2009), and most importantly, the need to connect, collaborate, and share resources with colleagues around the world. This general intensification, often referred to as “Big Data” science, is certainly true in biomedical research fields, such as neuroscience (Markram, 2013; Van Horn and Toga, 2013), and cyberinfrastructure has been proposed as a potential solution (Buetow, 2005). The efforts expended by many research groups in deploying cyberinfrastructures have unquestionably led to the development of successful new research methodologies. Neuroimaging platforms and applications have emerged that address common issues using drastically different approaches; from programmatic frameworks (Gorgolewski et al., 2011; Joshi et al., 2011) to advanced workflow interfaces, abstracting technological decisions away from users to various degrees (Rex et al., 2003; Olabarriaga et al., 2010). These applications excel in addressing different aspects of the problem; workflow building, leveraging data or compute grids, data visualization, collaborative elements (topic reviewed in Dinov et al., 2009). However, as these technologies are often strongly rooted in local requirements, they tend to form application and infrastructure “silos,” not easily adaptable to needs other than those for which they were originally conceived. Therefore, while the global nature of current scientific collaborations requires broader integration and platform interoperability, efficient integration of heterogeneous and distributed infrastructures across multiple technological administrative domains, in a sustainable manner, remains a major logistical challenge.

Over the past two decades, the evolution of neuroimaging research has led to the development of a rich array of data processing tools and complete analysis pipelines (exhaustive listing on the online NITRC^1^ repository). However, many of these tools remain unintuitive to the average researcher, as they require a solid understanding of advanced computer systems and display drastically differing underlying philosophies, which limits their potential for growth and adoption. They often require familiarity with command line and scripting techniques, long lists of configuration parameters and knowledge of how to properly prepare data for use as input. Manually processing heavier loads requires skills for data transfers and submission of analysis jobs to remote HPC sites in addition to a solid understanding of the scheduling software environment and policies used at each site. Furthermore, properly scaling these operations for large multi-site projects requires skills beyond all but the most technical research teams. Usability issues such as these lead to poor adoption of standards for tools and techniques, sub-optimal usage of resources, and immense amounts of replication and overhead cost. This alone represents a sufficient motivation to promote usage of common tools deployed in shared controlled environments where provenance details of each action are carefully recorded to ensure the reproducibility of results (Mackenzie-Graham et al., 2008).

The CBRAIN platform (http://www.cbrain.mcgill.ca) is a web-based, collaborative research platform designed to address the major issues of Big Data research in a single consistent framework. CBRAIN was conceived at a time when the question was no longer of creating resources such as HPC clusters and data repositories, since they already existed. Rather it was of creating a platform to leverage currently existing resources in a way that would best benefit the research community at large. Our primary objective was to build a user-friendly, extensible, integrated, robust yet lightweight collaborative neuroimaging research platform providing transparent access to the heterogeneous computing and data resources available across Canada and around the world. These key goals carry significant challenges. To address them, CBRAIN was designed with the following guidelines:

- Convenient and secure web access (no software installation required)
- Distributed storage with automated, multipoint data movement, and cataloging
- Transparent access to research tools and computing (HPC)
- Flexibility to adapt to extremely heterogeneous computing and data sites
- Full audit trail (data provenance) and logs across all user actions
- Lightweight core components, low requirements for deployment and operation
- Scalability (no architectural bottlenecks)
- Maintainability and sustainability by a research-based team
- Full ecosystem security and monitoring

The development of this type of integrated platform required addressing the aforementioned problems as they manifest themselves in brain imaging research. For example, pipeline tools are often built with hard-coded interactions to a particular cluster scheduling system, showing little understanding of proper HPC usage or consideration for site-to-site portability. This leads to a massive waste of resources as the generated workloads must be re-encapsulated for responsible use of public or shared HPCs. In addition, procedures and policies at various HPC sites, even within the same organization, can differ significantly, imposing additional burden on users and platform builders. Although sites may claim to use the same scheduling software, different scheduling policies may be implemented; queue limits and priorities vary, installed libraries and environment configuration vary, location and performance of various local storage may differ greatly.

In order to foster more flexible national and international collaborations, we seek to extend CBRAIN past these technological borders. CBRAIN was built in several layers, with a focus on ensuring tight coordination of the entire ecosystem: abstraction of extremely heterogeneous computing resources scattered over large distances; abstraction of remote data resources and a collaborative portal entirely accessible from a regular web browser where users can securely control and share, as desired, data, tools, and computing resources. In this paper, we will discuss how the above philosophy and guidelines have been implemented in CBRAIN and we will present the current deployment and usage of the platform within our neuroimaging community.

## Materials and methods

### CBRAIN overview

**Figure 1:**
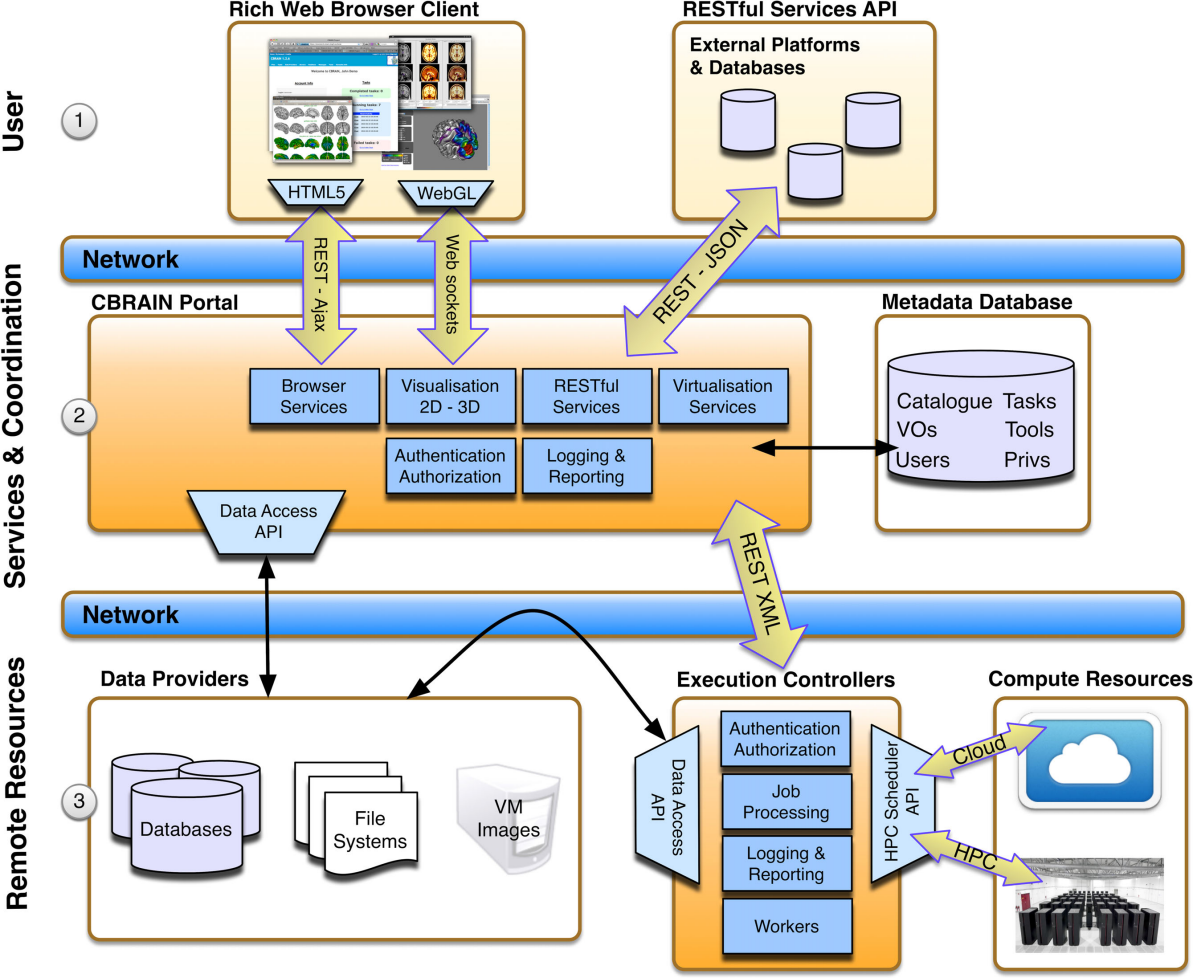
**CBRAIN architectural layers**. The top user layer (1) represents consumption of services through web browser clients or RESTful API. The central services and coordination layer (2) hosts CBRAIN portals that are responsible for providing services and business logic for requests from the top user layer and orchestration for the lower resource layer. The state of all model instances (users, VOs, tools, resources, catalog, privileges, etc.) is stored in the metadata database. In the lower remote resource layer (3) lays the data providers (scientific data servers, databases or virtual machine images, and tools repositories) and the execution controllers. Execution controllers have to be located at the computing sites on a node that has access to the system scheduler and cluster file systems. Note that data transfers between data providers and execution controllers are triggered by the coordination layer, but do not pass through this layer.

CBRAIN is a multi-tiered platform composed of three main layers (see Figure 1): (i) the access layer, accessible through a standard web-browser (for users) or a RESTful Web API (for applications or other platforms), (ii) the service layer which provides portal services for the access layer, the metadata database, which stores information about all users, permissions, and resources, and orchestration services for resource coordination (users requests, data movement, computing loads, jobs, data models,…), and finally (iii) an infrastructure layer consisting of networked data repositories and computing resources. An arbitrary number of concurrent data sources (data providers), computing sites (execution servers), and CBRAIN portals may co-exist, with only the metadata database as a central element for a given deployment. A data-grid mechanism with synchronization status tracking has been designed to avoid transfer bottlenecks and ensure scalability. Data transfers are coordinated directly from data providers by execution servers, ensuring that data are not transferred through the central service orchestration layer during operation, and that remote data providers are not overwhelmed by direct connections from processing nodes. Data visualization, being handled directly by a CBRAIN portal server, is the only major service that requires a data transfer to the central servers. This core flexibility allows a wide array of possible site setups. The simplest being the creation of a Virtual Site (also referred to as Virtual Organization or VO) and associated user accounts. These users will obtain access to CBRAIN shared storage and computing resources, but their data will remain private unless they explicitly decide otherwise. Sites can also integrate their own data providers and/or computing resources (again, shared or private). In addition to hosting private data providers and computing servers, a site may host its own CBRAIN portal within the walls of its institution and explicitly limit all operations to private local resources and private network. Such a configuration ensures a completely local handling of scientific data while at the same time benefiting from the advantages of the platform.

**Figure 2:**
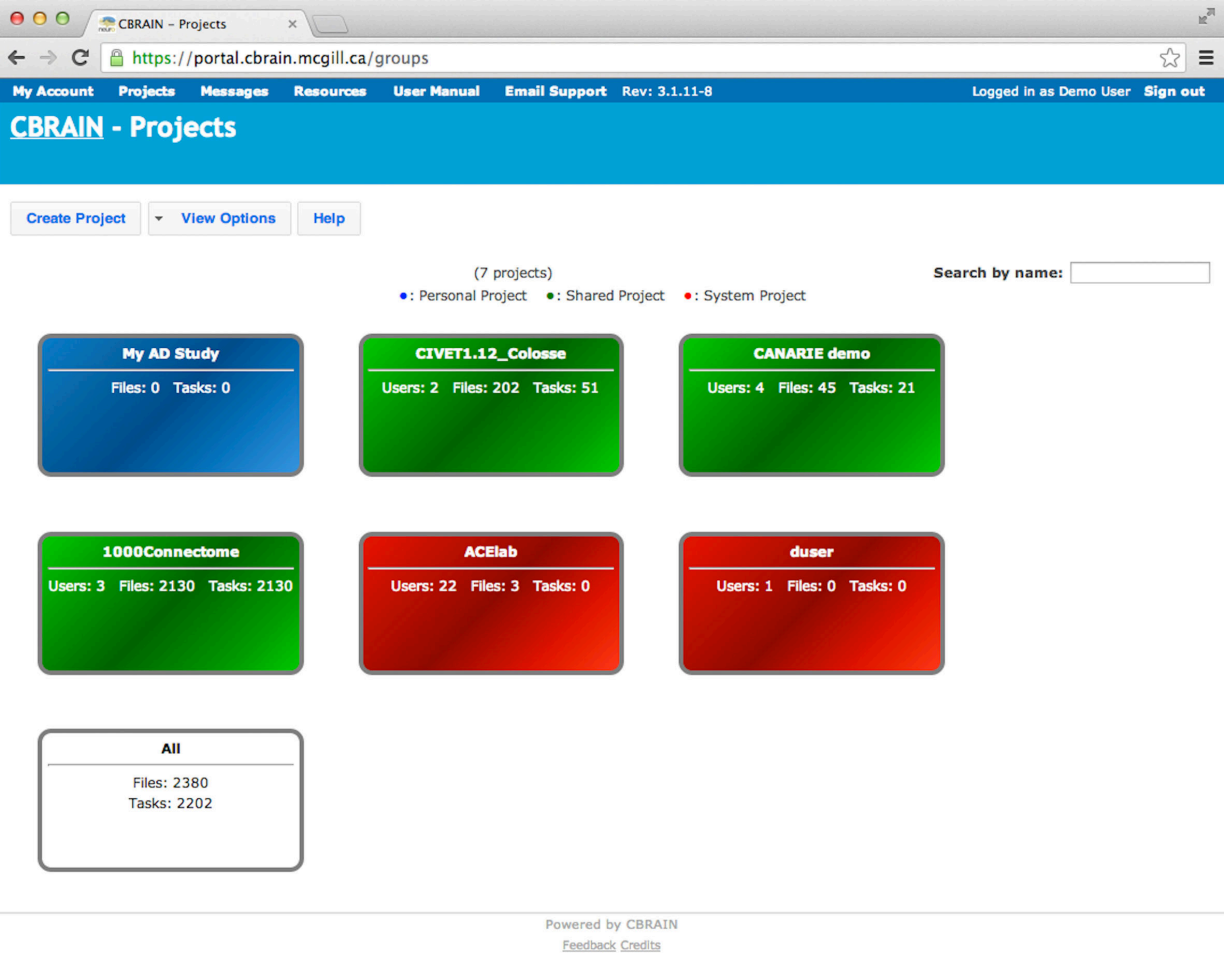
**CBRAIN portal: project view**. Authenticated users can see a representation of the various projects they own. Projects are color coded: blue for personal projects, green for shared projects, red are default user or site projects, and white allows access to all files owned by this user.

**Figure 3:**
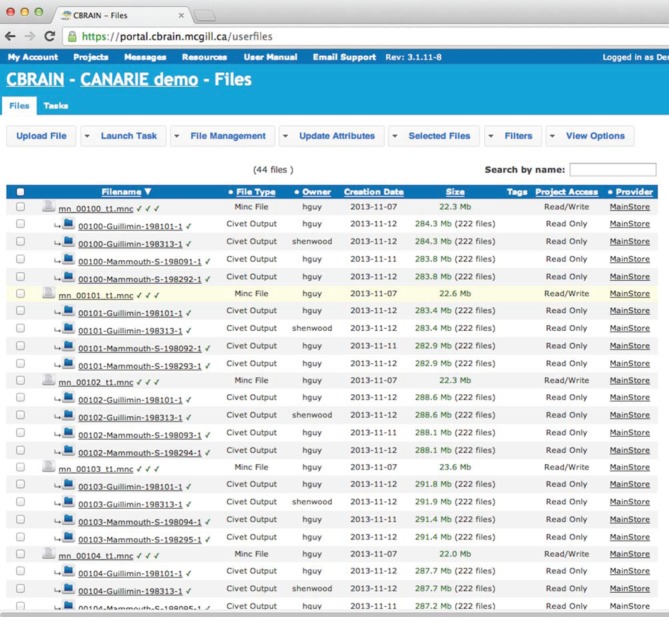
**CBRAIN portal: file view**. The file view is the main control space where users can manage file or file collection properties (name, privileges, project, tags, type, physical location), filter and select input files based on any property and select a tool for a given task. Web uploads and downloads can be performed through this page, although private data providers or SFTP transfers are preferable for large data. Synchronization information of a file or collection over various caches and data providers is indicated by a symbol next to the file name.

**Figure 4:**
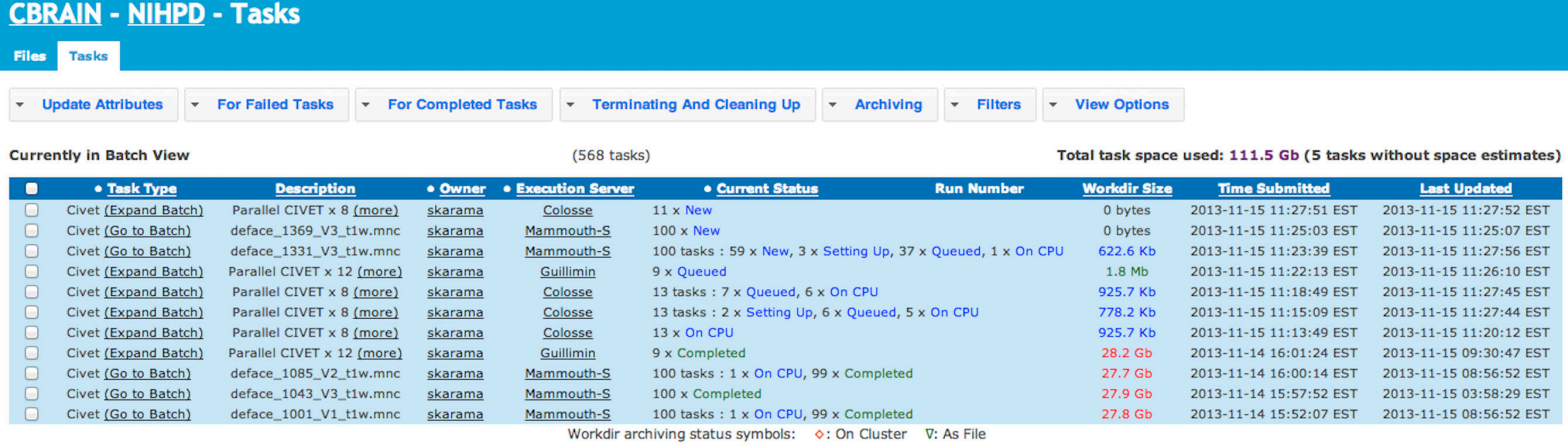
**CBRAIN portal: tasks view**. The tasks view allows monitoring of task progress, if desired. In this example, the CIVET pipeline has been launched on 1082 MINC files. This workload was split in 568 tasks on 3 different computing sites. CBRAIN has automatically packaged the jobs in proper task units for each execution server. *Colosse* provides full node scheduling with 8 cores per node (Parallel CIVET x8), *Guillimin* has the same type of scheduling, but with 12 cores per node (Parallel CIVET x12), while *Mammouth-S* provides per core scheduling. Although the user has full control of the tasks across the various sites, this is completely optional and transparent. Once jobs are completed, results are automatically transferred to the selected project.

**Figure 5:**
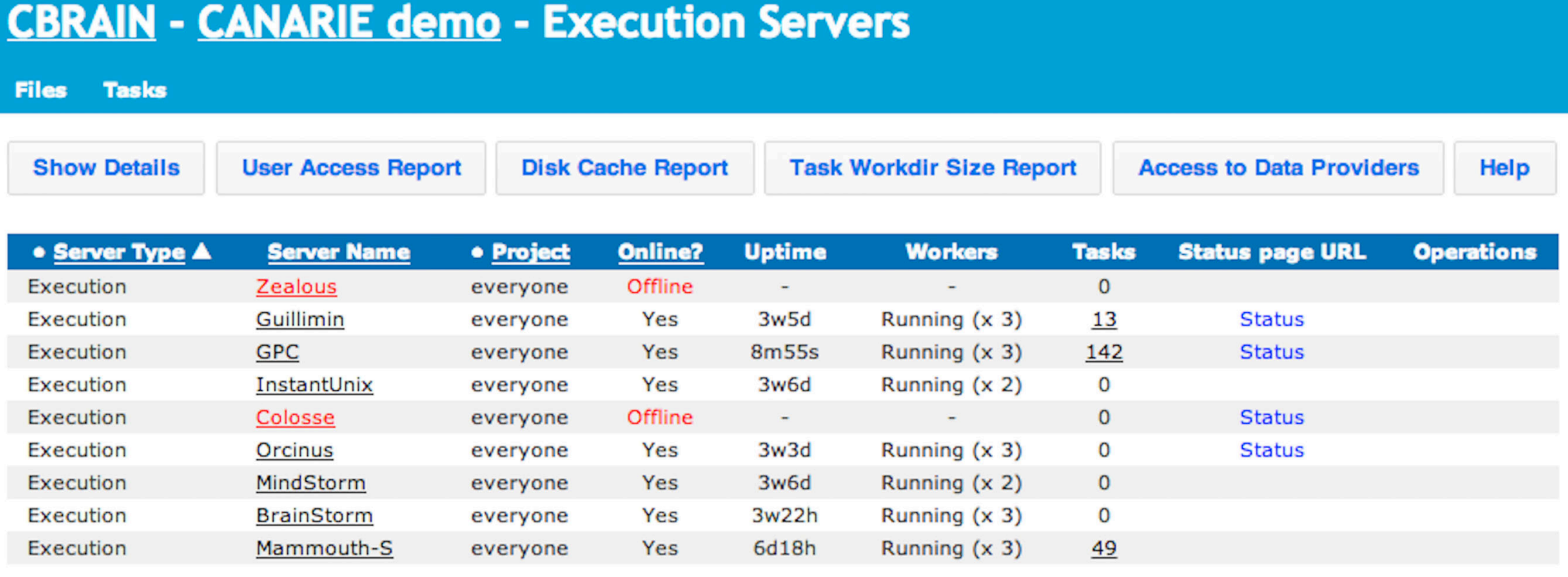
**CBRAIN portal: execution servers view**. This view allows users to see which computing resources are available for his/her use and their real-time status. Users can also obtain reports on tool access, cache and data provider utilization, and archived work directories. Administrative users can control group access and put the resource online or offline for CBRAIN users.

**Figure 6:**
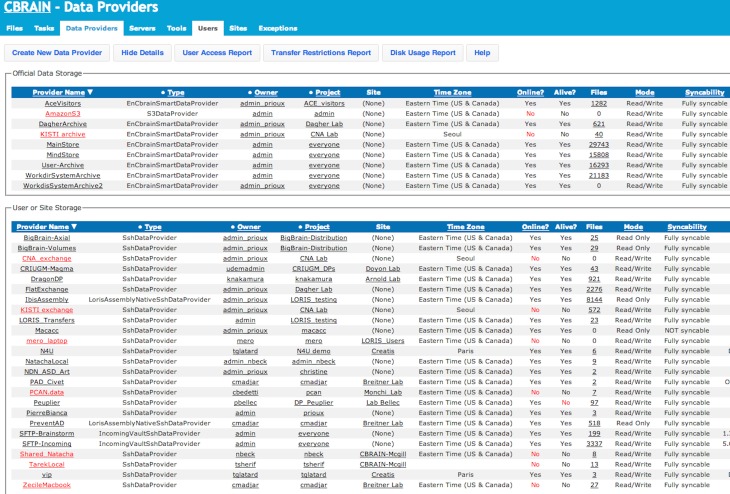
**CBRAIN portal: data providers view**. This view, presented from an administrative account, shows both the real-time status of official CBRAIN data storages (top) and user created storage (bottom). Main information shown: type of connection, project, owner, time zone, number of registered files and/or file collections, read/write mode, and synchronization mode. Reports for group access, transfer restrictions, and disk usage are available.

The CBRAIN web portal allows users to authenticate and manage their data and analyses. It also provides several advanced visualization tools for exploring results and performing quality control. The main components of the user environment are shown in Figures 2–4; namely the project view, file view and task view. Data is organized in user-created personal or shared projects (Figure 2). The file view (Figure 3) shows all data files and associated results registered in a selected project from all physical storage locations. Once files or collections are registered in the platform, users can filter, manage, tag, move, and share them across physical locations through a graphical user interface and without having to manage authentication, hostnames, and paths. The same principle applies to tool usage; the user simply selects a set of files and a tool, fill a tool parameter form and launches jobs to be executed remotely. All data transfers, environment setup, scheduler interactions, and monitoring are handled behind the scenes by CBRAIN. Current tasks (sets of computing jobs from various user operations) can be monitored, managed, and troubleshot, if desired, from the task view (Figure 4). Once completed, output files appear in the file view as children of the input files (see Figure 3). Complete audit trails (provenance) are available for all user actions: logins, file movement and transfers, task parameters, tool versions and logs, resources used, work directories. Links between input files (parents), compute jobs and output files (children) are maintained to allow convenient event browsing when doing post-analysis investigation. Resource views show the status of all data and computing resources accessible to the user (Figures 5, 6). The portal also provides a RESTful Web API that exposes CBRAIN functionality to other systems (Figure 1). This API allows decoupled cross-platform interoperability; any authorized system may authenticate, exchange data, and launch jobs on CBRAIN.

Access policies that regulate the use of CBRAIN-mediated resources for any given project are beyond the scope of this report since access restrictions do not arise from technical limitations. CBRAIN provides flexible capabilities to enforce data access and transfer policies on any computing resource, data source or tool, limiting access to specific users or groups and preventing actual scientific data or services to cross specific boundaries (such as institutional networks) whenever required.

### Distributed computing

Computing servers or HPCs connected to CBRAIN run a lightweight execution server. The execution server awaits requests for job submission, performs any setup required by the HPC site and then forwards the job submission request and parameters to the HPC's scheduler. The first challenge faced by CBRAIN was to manage the heterogeneity of these compute resources. Frequently, computing sites are built independently using different architectures, cluster job scheduler software, UNIX environments, storage setups, and overarching usage policies. Developing a centralized point of access that would be reasonably easy to use meant these differences in system architecture had to be overcome in a way that is invisible to the user. CBRAIN addresses this problem in several abstraction layers. The first layer is the Simple Cluster Interface in Ruby (SCIR), a custom library developed in-house.

SCIR was developed as a streamlined meta-scheduler to abstract scheduler differences away from the core platform. SCIR is a simple Ruby library that implements basic high-level functionality required to query, submit, and manage jobs to a given cluster job scheduler. It is implemented with a plugin architecture that makes it easily adaptable to new environments. New grid environments are supported by creating simple SCIR subclasses in Ruby implementing the base SCIR API. SCIR subclasses currently implemented provide support for current and legacy versions of SGE, PBS, Torque, MOAB, and several custom managers and direct UNIX environments.

CBRAIN execution servers simply run in a regular user account on a cluster head node. An execution server on a given HPC receives requests from the CBRAIN portal containing information about the requesting user, the location of data required for analysis, tools and parameters to use, and the data provider on which to store the results. The server can then synchronize the data to the HPC and make any preparation required by the tool or the HPC in order to successfully run the analysis. This can include creating work directories or setting up environment variables. The execution server then uses SCIR to optimize, convert, and submit the job requests to the local cluster scheduler. Once analysis is done, the execution server initiates transfers of the results to the data provider selected by the user. The execution server is configured through an administrative web interface where parameters such as scheduling type (by core or by node), number of cores per node, maximum queue occupancy, libraries and environment paths, and cache and scratch directories can be set. CBRAIN also performs meta-scheduling activities, such as monitoring jobs, performing failure recovery, optimizing, and re-packaging job loads to match different cluster environments and buffering excess jobs in a meta-queue when quotas are exceeded.

### Distributed storage

The CBRAIN data provider is an abstract model representing a data repository securely available to the platform from the Internet. Similarly to SCIR, the data provider is a programmatic API that abstracts away the details of specific types of data stores. The data provider defines a base class of uniform programmatic API methods for querying a file, transferring it, mirroring it and so on, and plugin Ruby classes implement the methods for a particular data store type, allowing CBRAIN to interact with it transparently. CBRAIN widely uses asynchronous data provider wrappers defined for rsync over SSH and SFTP protocols for connecting to data stored remotely. The choice of these tools and protocols does not represent file transfer methodology preferences but rather a pragmatic adoption of the mechanisms commonly supported by data and computing sites. Such mechanisms are also easily manageable by users (site administrators can create a new data provider with the web interface by pointing to the service and adding the CBRAIN public key in the proper account) for greater flexibility and extensibility. These automated grid-like methods have proven robust enough to connect CBRAIN to storage ranging from dedicated network file servers to smartphones. Cloud storage APIs for services such as Amazon S3 and Dropbox, are in the prototyping stage.

A distributed storage model does, however, make network performance a potential concern. CBRAIN makes heavy use of CANARIE's advanced research network^2^ and robust synchronization and caching mechanisms were built into the core platform to avoid unnecessary data transfers. The portal and execution servers maintain a local cache of the files that have been asynchronously transferred to them. Synchronization status for all data in all caches is maintained in the metadata database by CBRAIN. Resources will use cached versions of files until the version on the data provider changes, at which point all cached versions will be flagged as invalid. Resources caching invalid data will simply resynchronize with the data provider upon the next requested data operation. Users can manually trigger cache deletion of their data if desired. In addition, execution servers use a throttled data transfer model (Park and Humphrey, 2008). To avoid scalability issues, they initiate only a limited number of concurrent data transfer connections.

For most tasks, data stored on a data provider need never be transferred to the central CBRAIN server. Users can keep their data locally, CBRAIN will transfer it directly from their local stores to an HPC cache to run analysis, and then have the results transferred directly back to their data provider. If a lab or an institution has a private HPC with the proper tools connected to CBRAIN, the data need never leave their institution for processing. They can take full advantage of the abstraction provided by CBRAIN while maintaining full control over the location of their data. The only tools that may require that some data be sent to the CBRAIN portal server are the visualization tools as well as browser uploads and downloads for small datasets. To upload or download large datasets, CBRAIN offers SFTP services for users who do not have private data providers.

### Security

Users authenticate into the system by first logging into a private account. All communication between clients and the service middleware layer happens over a secure socket layer (SSL). Interactions between the middleware layer and remote resources occur through secure shell tunnels (SSH) with standard 2048 bits key encryption. As many resources used by CBRAIN are outside of our administrative domain, controlling exposure, and potential propagation of intrusions through intermediate machines is a fundamental security concern. CBRAIN uses an on-demand SSH-agent forwarding mechanism to create communication channels between portals, execution servers and data providers, sending all key challenges back to the service layer and closing all channels when not in use. In addition, CBRAIN is equipped with an SSH-agent locking mechanism. Unlocking requests are made by execution servers using a special key stored in the CBRAIN database. Tunnels are thus opened on demand, conditional to the establishment of the proper handshake and closed as soon as the transfer operation is complete. This has several advantages: it eliminates the risks associated with passwords or private keys located on any intermediate machines, it minimizes the duration of open tunnels and it allows platform administrators to carefully monitor whether the key challenges are associated with actual platform operations or possibly suspicious activities.

### Permission model

Access to all resources in CBRAIN is managed by three central concepts: users, projects, and sites. Users represent the account of a CBRAIN user. Once authenticated users are granted access to their environment and to any resources to which they have access. By default, users only have access to data they have added through their account. Ownership can be applied to any object within CBRAIN. This can include data, HPC jobs, projects, data providers, and HPCs. Ownership provides full read/write access: a user can rename, move, edit, or unregister any resources they own. On the other hand, if a user is associated to a shared resource through a site or a project, their access to the resource will depend on how it was configured by its owner.

Projects define shared access to resources. All data providers, data, execution servers, and tools in CBRAIN are associated with a project. Projects can have one or more users as members, and members of a given project will have access to the resources associated with it. This system is similar to group permissions in Unix-like operating systems. Users can create and manage their own projects, and by default the resources associated with these projects will be available only to the project's creator. A user can, however, invite other users to their projects, making it possible to share their data, tools, data providers, and execution servers with others.

A CBRAIN site represents a VO such as a laboratory, institution, or a distributed research group that wishes to have some control over how its resources are used in CBRAIN. A site will have users and projects associated with it, and one or more of those users can be given the role of site manager. A site manager has administration capabilities for resources associated with a given site. They can create and manage user accounts, projects, and other resources for their site. Essentially, a site creates an administrative subdomain in CBRAIN over which one or more local site managers can have control.

### Plugins and visualization tools

Once data has been processed, users often need to visualize their results. This can be for the purposes of performing quality control on a job that was run, or simply to explore the data in a meaningful way. In many processing-centric platforms, this would require a user to transfer large data sets to their computer and run locally installed visualization software. The CBRAIN portal, however, integrates visualization tools that allow users to explore their data in real-time through their web browser, with only the data necessary for the visualization being transferred to the client. At the most basic level, if a data set contains standard images or quality control related text, these can simply be made available for viewing through the browser. More complex visualization tools can be made available through CBRAIN's viewer plugin architecture, which associates file types with viewers. Formats viewable in CBRAIN currently include text, images, video, audio, MINC volumes, MNI 3D objects, and file types supported by Jmol (molecular structures). Display of most supported types involves simply using the appropriate HMTL element. CBRAIN does, however, provide more complex visualizers for MINC volume data and various surface file formats in the integrated BrainBrowser suite of web-enabled visualization tools (demonstration service available at https://brainbrowser.cbrain.mcgill.ca).

**Figure 7:**
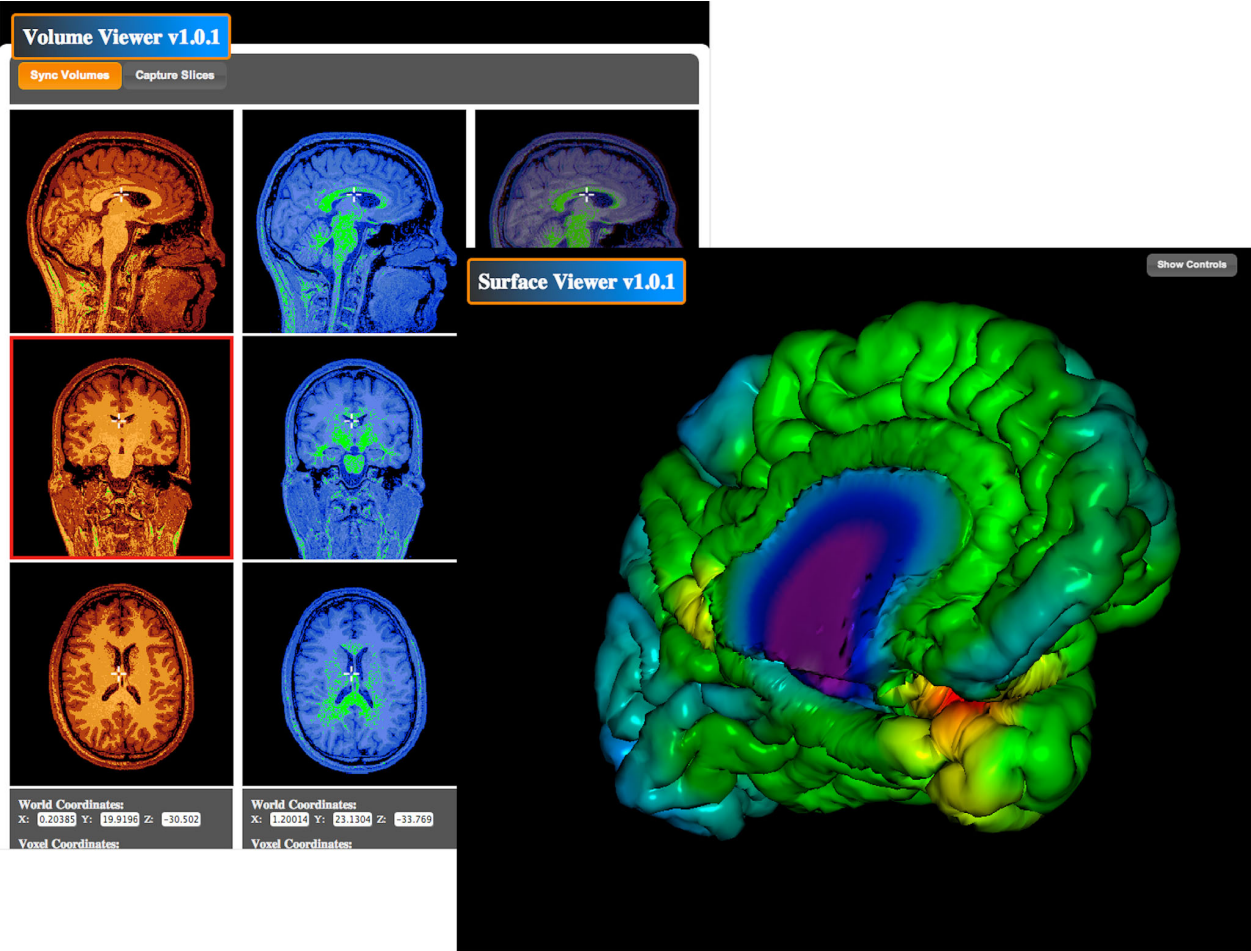
**BrainBrowser Surface and Volume Viewers**. BrainBrowser allows CBRAIN users to examine any MINC file volume or 3D object (such as surfaces from CIVET, Freesurfer, or Wavefront objects) directly within their web browser. This step enables to conveniently perform quality control, which is often critical before proceeding to further analysis or large data transfers, especially if format conversion steps have been applied.

The BrainBrowser Surface Viewer (Figure 7) is a web-based, real-time 3D surface viewer capable of viewing MNI Object, Wavefront Object, and Freesurfer ASC files. BrainBrowser allows users to view and manipulate 3D surface data in real-time. Color map data can be applied to surfaces, and color thresholds and opacity can be adjusted to ensure proper viewing. The BrainBrowser Surface Viewer is currently being used to provide web access to the MACACC data set (Lerch et al., 2006). The BrainBrowser Volume Viewer (Figure 7) is a web-based, slice-by-slice viewer for 3D MINC volumes. The Viewer provides three panels, one each for the sagittal, coronal, and transverse planes. Each panel displays a slice on a given plane at some position in the volume, and the user is allowed to navigate through the volume by moving the cursor within the volume. Four-dimensional fMRI data can be viewed by manipulating time sliders to view the data across time steps. Subjects can be viewed side-by-side and overlaid. Color maps and thresholds can be adjusted to optimize viewing.

### Technology used

CBRAIN components are implemented using Ruby on Rails^3^ (Bachle and Kirchberg, 2007), a widely used RESTful, Ruby-based framework, used by such sites as Github, Twitter, Shopify, Groupon, NASA, Hulu. Our core objective was to follow cutting-edge architecture and development strategies. The key to using Ruby on Rails in a distributed multi-component ecosystem like CBRAIN was streamlining the activities of the various layers and offloading any longer term processing to subsystems. This approach allowed us to take advantage of the built-in object-relational mapping (ActiveRecord) and RESTful nature of Ruby on Rails, while at the same time ensuring that the platform performs and scales elegantly. It also requires less development, hardware, multi-site setups, and operations personnel than common enterprise technologies such as frameworks based on Java. The portal uses Ruby Thin servers behind an Nginx load balancer and a MySQL database to track metadata pertaining to all resources. Using Ruby on Rails also allowed us to develop an agile methodology based on rapid iterations made with constant feedback from users.

CBRAIN development aims to use openly available tools and standards-compliant web technologies whenever possible. This ensures that development and distribution of the system can remain free and unrestricted. All browser interactions with CBRAIN occur over HTTPS and the web client uses standard HTML and CSS for the interface and jQuery^4^ and jQuery UI for behavior and theming. The BrainBrowser Volume Viewer uses the HTML canvas element for rendering, and the BrainBrowser Surface Viewer uses three.js^5^ for WebGL-based 3D rendering.

### Interoperability

CBRAIN exposes a RESTful Web API to allow interoperability with other platforms and database systems that want to take advantage of its capabilities. Requests are made to the same URLs used for the CBRAIN portal interface using standard HTTP methods (through SSL). The body of an API request can contain XML or JSON and the response will be an XML document representing the data requested. Wrappers for the CBRAIN Web API have been written in Java, Perl, and Ruby. Our usage of Ruby on Rails framework coding conventions ensures that all user interactions with the portal naturally map to RESTful API calls that return XML rather than HTML upon request. This greatly reduces the necessary work required to convert and support the API for cross-platform interoperability.

## Results

### Current deployment and use

CBRAIN has been in active production since 2009 and currently has over 200 users and 80 virtual sites, from 53 cities in 17 countries around the world. Operations are scaled on a yearly basis according to both the yearly computing allocation we obtain and the amount of user support our team can provide. The current production deployment of CBRAIN consists of 12 computing sites, totaling more than 100,000 CPU cores. The infrastructure model is hybrid, while many large clusters are shared national academic research resources (Table 1), others sites are institutional or completely private and available solely to CBRAIN. Of these sites, 7 are from the Compute Canada^6^ HPC network, 2 are international collaborator sites (Germany and South Korea), and 5 are small local research servers. This integration of heterogeneous resources was done without any new hardware purchases, and does not require administrative access or major changes to local system configuration on the part of the participating sites. Between 2010 and 2013 CBRAIN has launched in excess of 198,000 jobs and obtained an allocation of 13.7 million CPU core hours from Compute Canada alone. CBRAIN provides users with three central data providers, for a total of 80 TB of storage. Furthermore, several user-registered data providers exist as storage for specific projects or institutions. Although it fluctuates significantly, active data currently hosted on the central storage system provided to all CBRAIN users amounts to approximately 13.1 TB in over 100,000 datasets representing 8.4 million files (this does not include computing site caches or user-registered data providers).

**Table 1:** **CBRAIN high performance clusters and servers**.

**Machine name**	**Administrative domain**	**Location (city, country)**	**Number of CPU cores**
BrainStorm	McGill university	Montreal, Canada	24
Colosse	Compute Canada	Quebec City, Canada	7680
GPC	Compute Canada	Toronto, Canada	30,000
Guillimin	Compute Canada	Montreal, Canada	14,000
Judge	Jülich supercomputing center	Jülich, Germany	2472 + 412 GPU
Juropa	Jülich supercomputing center	Jülich, Germany	17,664
CBRAIN-CNA	KISTI	Seoul, Korea	80
Mammouth-S	Compute Canada	Sherbrooke, Canada	2112
Mammouth-P	Compute Canada	Sherbrooke, Canada	39,648
MindStorm	McGill university	Montreal, Canada	24
Orcinus	Compute Canada	Vancouver, Canada	9600
Zealous	McGill university	Montreal, Canada	24

List of servers and HPCs currently integrated in CBRAIN. All CPUs use standard x86-64 processor architecture and all operating systems are Linux based.

CBRAIN provides a wide variety of tools, from pre-processing and analysis pipelines to various file format converters for file types commonly used in neuroimaging research, including MINC, DICOM, NIfTI, and Analyze. Tool integration is prioritized according to the needs of our user community. CBRAIN's philosophy has been to focus on integrating, testing and properly maintaining and supporting tools and features directly requested by our researchers. The platform supports multiple tool versions and the version used in a specific analysis is maintained in the task and provenance logs. Among the most intensively used tools in CBRAIN is CIVET-CLASP (Kim et al., 2005), a processing pipeline for measuring cortical thickness, as well as performing other corticometric and volumetric functions. Components of the popular FSL^7^ (Jenkinson et al., 2012), MINC^8^, SPM^9^, and Freesurfer^10^(Reuter et al., 2012) tools have also been integrated. These types of tools are ideal candidates for CBRAIN integration as they are computationally expensive and generally complex to use for the novice user. Most neuroimaging tools have a relatively straightforward workflow, with job inputs and options following a linear sequence of events. However, some pipelines dynamically allocate jobs and dependencies in real-time depending on the inputs they receive. Such job loads have to be carefully analyzed and packaged to ensure optimal use of HPC resources. For example, CBRAIN uses a graph theoretic approach to serialize and parallelize the dynamic job loads of tens of thousands of jobs from NIAK, an fMRI pre-processing pipeline based on the Neuroimaging Analysis Kit for Matlab and Octave, described in Lavoie-Courchesne et al. (2012).

Cross-platform interoperability features have been implemented both in the context of our group's multi-center management system, LORIS (Das et al., 2011) and external collaborative efforts. As part of the “neuGRID 4 you” project (Frisoni et al., 2011), the CBRAIN Web API was consumed by the neuGRID and Virtual Imaging Platform (Glatard et al., 2013) services in Europe using the LONI Pipeline software (Rex et al., 2003). A CBRAIN module for the LONI Distributed Pipeline Server (DPS) was created to interact directly with the CBRAIN Web API. This type of collaboration positions CBRAIN as part of a global network of research platforms, enabling collaborations between users and allowing them to take advantage of the broadest set of services possible.

Although CBRAIN is a generic platform that can accept data and analysis tools from any discipline, its current focus is primarily on structural neuroimaging projects. For example, CBRAIN has been used in a study linking childhood cognitive ability and cortical thickness in old age where DICOM sets from 672 subjects of the Lothian Cohort 1936 were uploaded and registered in CBRAIN from a research group in Scotland, and shared with a group of Canadian researchers for pre-processing and analysis of cortical thickness (Karama et al., 2013). Other examples of initiatives actively using CBRAIN for typical MRI data pre-processing of large cohorts are PreventAD^11^, NIHPD^12^, NeuroDevNet^13^, ABIDE^14^, and 1000Brains^15^.

## Discussion

### Related work

The CBRAIN platform incorporates the key aspects of a grid middleware, namely security (Authentication, Authorization, Accounting—AAA), distributed file management, and job execution on multiple distributed sites. Grid middleware has received a lot of attention in the last 15 years (Foster and Kesselman, 2003), and resulting technologies and concepts are now used in large computing infrastructures such as the Open-Science Grid (Pordes et al., 2007), Teragrid (Catlett, 2002), and the European Grid Infrastructure (Kranzlmüller et al., 2010). CBRAIN is unique in the sense that it integrates all these functions in a single, consistent, lightweight, self-contained, independent framework that is therefore easily administrated and extended. For example, grid security usually relies on X509 certificates signed by trusted authorities, from which time-limited proxy certificates are generated, delegated to the services involved in the platform, and used to authenticate all user operations, for instance job execution and data transfers (Foster et al., 1998). In practice, this mechanism burdens users with the handling of certificates, restricts the range of usable technologies, generates user-specific errors, and complicates debugging. To avoid these issues, CBRAIN decouples user AAA from system AAA: users authenticate to the portal with straightforward login and password, while the portal handles data and computing authorizations, and then authenticates to the services using a single or a few group credentials. Such decoupled approach is being adopted more broadly by portals using so-called robot X509 authentication to infrastructure services (Barbera et al., 2009).

Distributed file management commonly consists of a logical layer providing a uniform view of physical storage distributed over the infrastructure. CBRAIN's file metadata contain similar information to that stored in grid file catalogs, for instance the LCG File Catalog (Baud et al., 2005) or the Globus RLS (Chervenak et al., 2009). However, CBRAIN's file transfer architecture notably differs from the main grid solutions: (i) its throttled data transfer model avoids overloading storage providers, a problem commonly observed in grid infrastructures and addressed in a similar way by the Advanced Resource Connector (Ellert et al., 2007) (ii) it caches files on the computing sites, a feature only provided in a few grid middleware and often implemented at the application level.

Job execution on multiple distributed computing sites is performed either by a meta-scheduler which dispatches jobs to the different sites (Huedo et al., 2001; Andreetto et al., 2008) or by pilot-job approaches provisioning computing resources with generic agents that pull tasks from a central queue when they reach a computing node (Frey et al., 2002; Brook et al., 2003). In neuroimaging, however, due to variations of software and/or libraries, the execution site often has to be controlled by the users to guarantee the correctness and reproducibility of computations (Gronenschild et al., 2012). This is why CBRAIN usually delegates site selection to the users, providing them historical information about queuing times. The matchmaking between tasks and resources, which involves elaborate resource descriptions when performed by a generic grid middleware (Andreetto et al., 2010), is done statically by CBRAIN administrators who map application versions to sites based on their knowledge of the infrastructure.

The decision to develop SCIR as a streamlined meta-scheduler to abstract scheduler differences away from the core platform was based on pragmatic cross-site deployment experience. Libraries with similar goals do exist, but they did not demonstrate enough agility and flexibility for the HPC landscape we faced. The DRMAA (Tröger et al., 2007) and SAGA (Jha et al., 2007) projects, from the Open Grid Forum Working Group, were just emerging standards at the time of the initial CBRAIN deployment. DRMAA is a universal scheduler API library that was used in earlier versions of CBRAIN. Unfortunately, from our experience, although the library defines a fairly complete low-level API, the modules that actually interact with the cluster job schedulers were found to leave certain scheduler versions unsupported and were not designed to be easily extended for interaction with in-house schedulers. Our objectives for low-footprint and flexibility run contrary to dictating scheduler requirements to a diverse array of computing sites, so we created a library suited to our specific needs.

A few other science-gateway frameworks exist to facilitate the building of web portals accessing distributed infrastructures for scientific computing (Marru et al., 2011; Kacsuk et al., 2012). These frameworks provide toolboxes of components meant to be reused in customized assemblies to build domain-specific platforms. To ensure performance and flexibility, CBRAIN developed its own custom portal, which allows fine-grained, optimized interactions with infrastructure services. Other similar leading platforms providing access to neuroimaging applications executed on distributed infrastructures are LONI (Dinov et al., 2010), neuGRID (Redolfi et al., 2009), and A-Brain (Antoniu et al., 2012). While sharing similar overall goals, each platform uses often radically different approaches and philosophy, allowing them to excel in specific niches. For example, LONI offers an advanced and flexible graphical workflow builder that has, to our knowledge, no equivalent in the field. Within CBRAIN, our team took the design decision of supporting only mature, validated workflows as needs arise from our community. CBRAIN users are free to launch any tools or pipelines they have access to, but cannot create and share an automated workflow using multiple tools, the way it would be done in LONI, without contacting the core team. This has the advantage of preventing failures and waste of resources and of enforcing staged validation and quality control, however it does limit the rate of automated workflow integration and flexibility for the users. NeuGRID has a strong remote desktop component capable of providing remote users with native data visualization applications (centralized approach), CBRAIN handles all visualization applications through web-based applications (decentralized approach). These two approaches to the same problem have different characteristics, while the centralized approach procures users with familiar applications in their native mode, supporting usage growth can require large infrastructure investments. The decentralized approach uses very light infrastructure to push modern HTML5 applications to large amounts remote clients, respecting the CBRAIN scalability philosophy, however these applications have to be web compatible or developed anew. The A-Brain platform has done extensive work on low-latency data-intensive processing by building an optimized prototype MapReduce framework for Microsoft's Azure cloud platform on the basis of TomusBlobs (Costan et al., 2013). In comparison, CBRAIN focused on a lightweight, flexible and low-footprint catalog and data grid mechanism that acts as a transparent interface for regular multi-site batch-type projects. While it is clear that the CBRAIN grid cannot move and process multi-terabyte studies with the same ease as A-BRAIN, our goal was to ensure that all user sites can integrate securely in our grid their own data repositories with a minimum of requirements. This leads to a mix of faster and slower storage segments, which CBRAIN manages asynchronously with its caching mechanism. Most of our large imaging projects, with thousands of subjects representing hundreds of gigabytes of data can be processed as-is with the CBRAIN grid. Some multi-terabyte, data-intensive projects, such as our 3D histological reconstruction (Amunts et al., 2013), required special infrastructure for processing and visualization.

The modular plugin approach used to develop many of CBRAIN's components makes the platform easily extensible. New data providers, execution servers, visualization tools and other components can be added to the platform with a minimal investment of time and effort. On a deeper level, a small investment in development time can extend the base data provider and SCIR APIs to allow compatibility with new types of storage and cluster management. As an example, our team has begun experimenting with the integration of Amazon's S3 cloud as a data provider. CBRAIN as a meta-scheduler does more than provide a uniform API to the heterogeneous scheduling of various sites; it handles maximum queue allocations, node vs. core scheduling, max load per node, specific environment variables, caches locations, and data transfer tools/protocols on a per site basis. The platform excels at bridging the gap in common standards between existing cyber-infrastructures, providing transparent access to grids, public HPC sites, and private infrastructure through a single common framework.

### Future work

We are prototyping methods to extend the job model to accommodate the provisioning of Virtual Machines (VMs) on HPC and Cloud infrastructure. Thanks to the flexible and integrated development of CBRAIN components, these extensions can reuse several of CBRAIN's core services. For instance, the meta-scheduler is used to launch VMs from disk images equipped with application tools which are simply stored on data providers and handled by the CBRAIN data management system. Executing tasks in VMs facilitates the deployment of tools on classic HPC clusters, enables the exploitation of clouds, and ensures a uniform computing environment across heterogeneous infrastructures. Deployed VMs are seen by the platform as computing sites, opening possibilities for finer cross-site load balancing. This increased mobility across traditional batch HPC sites and actual clouds will allow us to further leverage resources from these two types of services.

Moving forward, priorities for the platform include further development and refinement of the Web API to allow other systems to take advantage of the services offered by CBRAIN. There are plans to extend CBRAIN into fields other than neuroimaging, such as epigenomics and the humanities. The platform itself is generic, meaning that in principle it should be usable in any domain that requires computationally expensive processing of large data sets.

### Obtaining and accessing CBRAIN

The core CBRAIN codebase will be made available as an open source project in mid-2014. Please refer to the NITRC site for instructions (https://www.nitrc.org/projects/cbrain). Trial CBRAIN accounts can also be obtained upon registration (https://portal.cbrain.mcgill.ca). For any registration or source code access questions, our group can be contacted at cbrain-support.mni@mcgill.ca.

### Conflict of interest statement

The authors declare that the research was conducted in the absence of any commercial or financial relationships that could be construed as a potential conflict of interest.

## Footnotes

^1^http://www.nitrc.org

^2^http://www.canarie.ca

^3^http://rubyonrails.org

^4^http://jquery.com

^5^http://threejs.org

^6^https://computecanada.ca

^7^http://fsl.fmrib.ox.ac.uk/fsl/fslwiki

^8^https://www.nitrc.org/projects/minc

^9^http://www.fil.ion.ucl.ac.uk/spm

^10^http://freesurfer.net

^11^http://www.preventad.com

^12^http://pediatricmri.nih.gov

^13^http://www.neurodevnet.ca

^14^http://fcon_1000.projects.nitrc.org/indi/abide

^15^http://www.fz-juelich.de/inm/inm-1/EN/Forschung/1000_Gehirne_Studie/1000_Gehirne_Studie_node.html
